# Offspring Number Does Not Influence Reaching the Disability’s Milestones in Multiple Sclerosis: A Seven-Year Follow-Up Study

**DOI:** 10.3390/ijms17020234

**Published:** 2016-02-12

**Authors:** Emanuele D’Amico, Carmela Leone, Francesco Patti

**Affiliations:** Department of Neurology, University of Catania, Multiple Sclerosis Center, Policlinico G. Rodolico, Via Santa Sofia, 78 Catania 95123, Italy; emanueledamico82@gmail.com (E.D.); carmelaleone84@yahoo.it (C.L.)

**Keywords:** multiple sclerosis, parity, disability

## Abstract

Objectives: data on pregnancy long-term effects on multiple sclerosis (MS) course are still controversial; whether experiencing more than one pregnancy exposes one to risk of the disability‘s accrual is still unknown. We investigated differences existing in terms of disability progression among women with MS (wwMS) who had one or more children after their MS onset. Methods: Monoparous and multiparous wwMS were enrolled from the Catania MS Center, Italy, in a monocenter retrospective study. A Cox proportional hazards model was used to examine the effect of the number of parities on time from MS disease onset to EDSS 4.0 and 6.0. The study protocol was approved by the local Ethical Committee. Results: during the seven years of observation, 32.1% and 23.2% of the monoparous group reached expanded disability disease status (EDSS) 4.0 and 6.0 respectively, compared to 13.3% and 3.3% of the multiparous group (*p* = 0.057 and *p* = 0.017; respectively). The Kaplan–Meier curve analysis showed no statistically-significant differences between the two groups in reaching the two milestones. The multiparous group showed a longer time to reach the EDSS 4.0 (3.5 *vs.* 2.6 years, log-rank 0.57, *p* = 0.45). The Cox regression analysis showed that the EDSS at the time of first pregnancy (Exp(B) 9.4, CI 4.5–19.7, *p* < 0.001) and the time from MS onset to first pregnancy (Exp(B) 0.96, CI = 0.93–0.98, *p* < 0.05) were significant predictors of reaching the EDSS 4.0, whereas a model including only the EDSS one year after the first pregnancy significantly predicted (Exp(B) value of 6.4, CI 2.6–15.4, *p* < 0.001) the reaching of EDSS 6.0. Conclusions: Our results suggest that experiencing more than one pregnancy could not convey a different clinical outcome in wwMS. Further research is needed to confirm our results.

## 1. Introduction

Multiple sclerosis (MS) is an immune-mediated disease that leads to demyelization and axonal loss in the central nervous system. MS strikes more frequently women than men and typically occurs during childbearing age [1]. Pregnancy was shown to influence MS disease activity, by a decrease in the relapse rate, especially during the third trimester. However, an increase in relapses within the first months postpartum leads the relapse rate to the pre-pregnancy baseline status [2,3,4,5]. An association between both pregnancy and a higher number of offspring with a decreased risk of MS conversion in women with a first demyelinating event was described [5]. Several theories were developed about the protective effects of pregnancy on MS disease activity and progression. The anti-inflammatory effects of sex hormones, such as estrogen and progesterone (increased during pregnancy, above all in the third trimester), could be responsible for the beneficial effects on MS [6,7,8,9].

In the last few decades, much effort was made in determining whether pregnancy has long-term effects on the disease course, above all on the disability’s accrual [9,10,11,12]. Yet, the results are still controversial. The same is about any difference in terms of the number of pregnancies and the risk of long-term disability [12,13]. Because MS strikes young women during their childbearing age, many women with MS (wwMS) are concerned about the consequences of the childbirth and whether their maternal experience could be even repeated in more than one pregnancy [13].

We aimed to study whether any differences exist in terms of the disability’s accrual among wwMS who had one child and wwMS who had more children after MS onset.

## 2. Results

**Figure 1:**
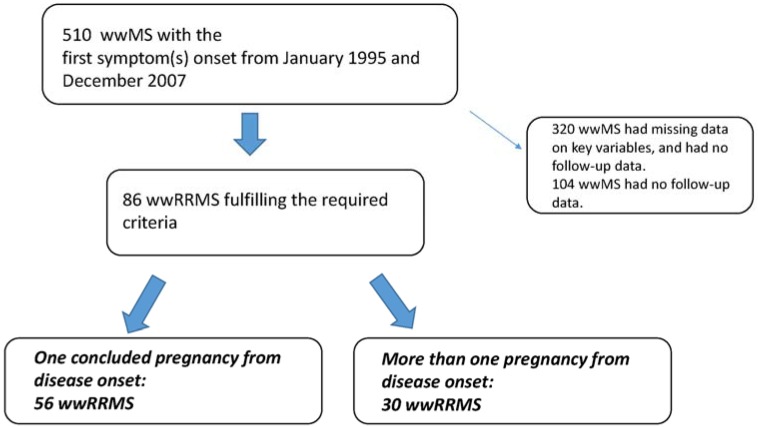
Selection flow chart. wwRRMS, women with relapsing-remitting multiple sclerosis.

From January 1995 and December 2007, a convenient sample of 510 wwMS was selected from a sample of 827 people with RRMS admitted in our clinic MS database iMed^©^. Out of 510, eighty-six wwMS fulfilled the required criteria to be included in the present study. Fifty-six (65.1%) wwMS experienced one pregnancy from the disease onset (monoparous group), while 30 wwMS had more than one pregnancy from the disease onset (multiparous group) (see Figure 1). Table 1 shows the demographic and clinical characteristics for the two groups. The two groups were significantly different for the age at the first pregnancy (31.0 *vs.* 28.5, *p* < 0.05), the number of relapses one year before the first pregnancy (1.1 *vs.* 0.6, *p* < 0.05) and for EDSS at time of the first pregnancy. The percentage of wwMS who breastfed was not significantly different between the two groups. Out of 56 monoparous wwMS, 23 (41.1%) switched drug treatment after the first pregnancy compared to four (13.3%) wwMS in the multiparous group (chi-square 6.9, *p* < 0.05). Table 2 shows the clinical outcomes reported during the follow-up period after the first pregnancy in the two groups.

**Figure 2:**
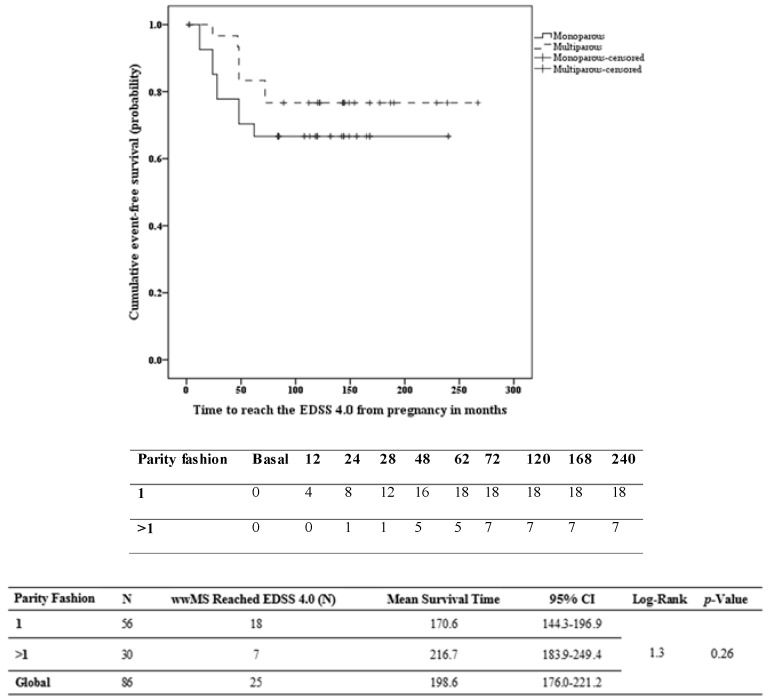
Kaplan–Meier survival analysis of the time to reach EDSS 4.0 according to the parity status. CI, confidence interval; EDSS, expanded disability status scale.

**Figure 3:**
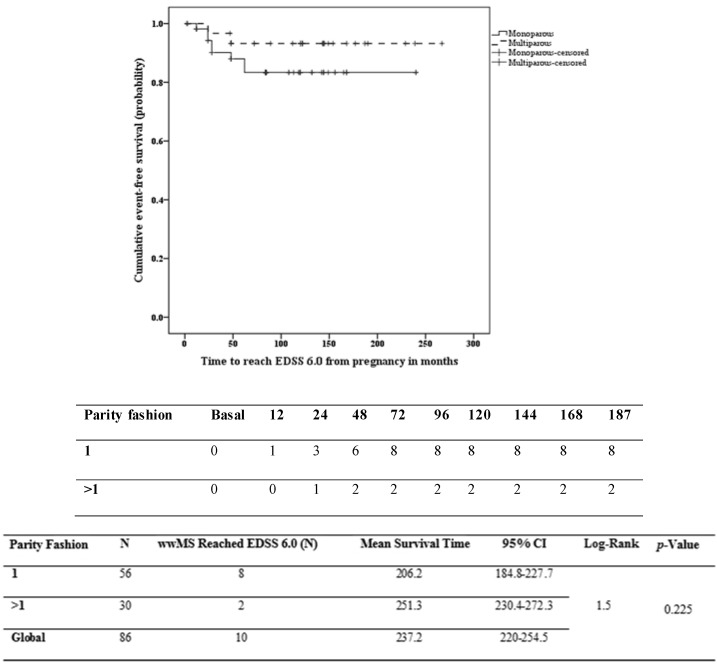
Kaplan–Meier survival analysis of the time to reach EDSS 6.0 according to the parity status. CI, confidence interval; EDSS, expanded disability status scale.

Survival curves between two groups showed no difference between multiparous wwMS (216.7 months, CI = 183.9–249.4 months) and monoparouswwMS (170.6 months, CI = 144.3–196.9 months) in reaching the milestone EDSS 4.0 (see Figure 2). No significant differences were found between the two groups in reaching the EDSS 6.0: monoparous206.2 months, CI = 184.8–227.7 months, *vs.* multiparous 251.3 months, CI = 230.4–272.3 months (see Figure 3). Low VIF values (<2.5) were observed between the independent variables, indicating that there was no interfering level of multicollinearity. The proportional hazard assumption was not violated as determined by log-minus-log plots. Cox proportional hazards analysis allowed for investigating the effect of parity on the risk of reaching EDSS 4.0 and 6.0, respectively, while adjusting for confounding factors.

**Table 1:** Demographic and clinical characteristics (mean ± SD) of the two groups based on the number of pregnancies.

Demographic and Clinical Characteristics	Monoparous *n* = 56	Multiparous *n* = 30	*p*-Value
Age (y, mean ± SD)	42.5 (38–45)	42.5 (38.7–48)	ns
Number of total pregnancies	1.0 ± 0	2.1 ± 0.3	<0.05
Age at the first pregnancy (y, mean ± SD)	31.0 ± 5.0	28.5 ± 3.7	<0.05
Lactation yes (%)	38 (67.8)	16 (53.3)	ns
Time from onset to the first pregnancy (months)	56.5 ± 45.6	51.6 ± 40.5	ns
Number of relapses one year before the first pregnancy	1.1 ± 0.8	0.6 ± 0.7	<0.05
EDSS at the first pregnancy *	1 (1.4)	1 (0)	<0.05
Mode of delivery (C-section yes, %)	14 (25)	5 (17)	ns
Child weight at birth	3.3 ± 0.9	3.2 ± 1.1	ns
Perinatal complication at first pregnancy	1	no	–

y = years; EDSS = expanded disability status scale; * median (IQR range), ns = not significant.

**Table 2:** Clinical outcomes reported during the follow-up period after the first pregnancy. Data are the mean ± SD for the two groups based on the number of pregnancies.

Clinical Outcomes	Monoparous *n* = 56	Multiparous *n* = 30	*p*-Value
Number of wwMS who reached EDSS 4.0 (%)	18 (32.1)	7 (23.3)	ns
Number of wwMS who reached EDSS 6.0 (%)	8 (14.3)	2 (6.6)	ns
EDSS 1 year after the first pregnancy	2.2 ± 1.4	1.4 ± 0.7	<0.05
EDSS at last follow-up	2.7 ± 2.0	2.1 ± 1.5	ns
Relapses during the first pregnancy	0.2 ± 0.5	0.0 ± 0.2	ns
Relapse of relapse 1 year after the first pregnancy	1.1 ± 0.9	1.0 ± 0.8	ns
Number of relapses at the last follow-up (after 7 y)	2.4 ± 2.7	2.7 ± 2.6	ns
Number of wwMS who switched therapy	23 (41.1)	4 (13.3)	<0.05

wwMS = women with relapsing-remitting MS; EDSS = expanded disability status scale; y = years.

Regarding the relationship between the covariates and the survival time to event EDSS 4.0, the significant predictors included in the model were the EDSS at the time of first pregnancy (Exp(B) 9.4, CI 4.5–19.7, *p* < 0.001) and the time from MS onset to first pregnancy (Exp(B) 0.96, CI = 0.93–0.98, *p* < 0.05). The hazard of reaching the EDSS 4.0 is reduced by 4% for each month a wwMS has lived without being pregnant since the MS onset. The hazard for a wwMS who has lived without being pregnant for one year since MS onset is reduced by 21.7%. See Figure 2.

In the analysis of the relationship between the covariates and the survival times to event EDSS 6.0, the only independent variable retained in the model was the EDSS one year after the first pregnancy with an Exp(B) value of 6.4, CI 2.6–15.4, *p* < 0.001, that is the hazard of reaching the EDSS 6.0 was 6.4-times greater for each EDSS point more at the time of first pregnancy. See Figure 3.

Parity fashion intended as mono- or multi-parity did not significantly contribute to the model. Time-to-event analysis did no yield significant differences between monoparous and multiparous wwMS.

## 3. Discussion

The offspring number does not influence the reaching of major disability milestones in wwMS. Multiparous wwMS took a longer time to reach EDSS 4.0, compared to monoparous wwMS, but this was not significantly different. The multiple regression analysis showed that only the disability level assessed at the time of first pregnancy together with the time elapsed between the MS onset and the offspring number had a predicting role in reaching the milestone EDSS 4.0, while the EDSS one year after pregnancy was the predictor of reaching the milestone EDSS 6.0. Even the proportion of patients who reached EDSS 4.0 did no differ significantly between groups. Our data hamper us in confirming previous evidence that number of pregnancies may convey a favorable disability prognosis in MS [14,15,16]. Previous studies have been conducted to investigate the role of pregnancy on disability progression, supporting that parous wwMS had a lower risk than nulliparous wwMS in reaching EDSS 4.0 and 6.0 [10,12,17]. To our knowledge, this is the first study conducted to evaluate whether, within “parous” wwMS, the number of pregnancies (“offspring number”) could be associated with the long-term disability status.

The influence of pregnancy in MS course have long been described in literature, but most of the data are reporting on short-term observation [18,19]. Ponsonby *et al.* [5] (investigating subjects at their first demyelinating event) found a reduced risk of developing definite MS as the number of pregnancies increased, and D’hooge *et al.* [14] showed an effect of parity in RRMS, but not in the progressive onset types of MS. The Pregnancy and Multiple Sclerosis (PRIMS) trial reported the data regarding pregnancy and the two-year post-partum follow-up period. A significant reduction of relapses during pregnancy (70% in the last trimester) was observed, with a sharp rebound to 70% above pre-pregnancy relapse rates during the first three months postpartum [3,4]. Recently, Teter *et al.* [12] showed that during a mean follow-up period of 5.6 years after delivery, the parous wwMS take a longer time to reach a disability milestone (the use of a cane, EDSS of 6.0). However, other studies showed no effect of parity on disability, even if the follow-up periods tended to be inconsistent or too short (3–12 months postpartum) [10,18,19]. In a small three-year prospective study, Worthington *et al.* [18] reported no significant differences between relapse rates, severity of relapses or MS disability at three years postpartum. A mean follow-up period of 10.3 years post-pregnancy in 32 wwMS showed that pregnancy exposure did not lead to increased disability [10]. Moreover, other prospective investigations described that it is unlikely that pregnancy and childbirth have an influence on the long-term prognosis for MS, that is offspring number might have no influence on the risk of secondary progression [20,21].

In the recent decade, pregnancy-related issues in MS have received growing interest. Until the end of the 1990s, wwMS were discouraged from having children owing to a biased belief that pregnancy would worsen the disease course. Since the first large prospective study in 1998, counseling of wwMS has changed radically, and many wwMS have attained their desire of motherhood. We have to bear in mind that our cohort enrolled wwMS since January 1995, that is a period when pregnancy was deemed dangerous for pregnancy outcome and a contributing factor for exacerbation of MS. This could justify why in our cohorts, wwMS with low disability and few relapses, may be more prone to get pregnant than one with a more active disease even if the median EDSS was still very low (median 1.0), even in the monoparous group (despite the significant difference with the multiparous group).

## 4. Methods

A monocenter, retrospective review of prospectively collected data was conducted. All wwMS were enrolled from the cohort of Multiple Sclerosis Center of Catania, Italy. Data were recorded according to the computerized and standardized protocols (European database for MS; EDMUS Coordinating Center Lyon, France and iMed^©^software’s; Serono International SA, Geneva, Switzerland). Only wwMS with at least one pregnancy event after the clinical onset of the disease (intended as the first symptom of the disease) were included. WwMS were divided into: (1) the monoparous group (wwMS having borne only one child after disease onset); and (2) the multiparous group (wwMS who have borne more than one child after disease onset). WwMS who experienced concluded pregnancies before the MS onset were excluded.

The diagnosis of clinically-defined MS was done according to the McDonald, Lublin and Poser criteria [22,23,24]. The relapsing remitting (RR) phenotype was defined as a history of relapses and remissions without progressive deterioration at the beginning of the disease [24]. Only wwMS having a period of observation of at least 7 years from the first concluded pregnancy (at the time of the last available follow-up visit) were finally included. We excluded wwMS without a concluded pregnancy (abortion).

At the first follow-up visit, the following data were collected: age at the first pregnancy, lag time from disease onset to the first pregnancy, the mode of delivery, offspring weight at birth, breast-feeding status, the health status of the child and the occurring of any perinatal complication; disability status was assessed by stable expanded disability disease status (EDSS, [25]) (that is, an EDSS evaluation far from any neurological relapse, but within the year previous to the index date). A longitudinal prospective collection of clinical data was performed from the time of the first admission at our MS center until at least seven years after the first pregnancy. The MS-related clinical outcome collected at each follow-up every sixmonths included: relapse occurrence, a neurologic visit with EDSS evaluation and the status of disease-modifying and symptomatic treatments.

We chose two known disability outcomes as disability milestones: (1) a score of 4.0 on the EDSS, indicating “fully ambulatory without aid, up and about 12 h a day despite relatively severe disability, able to walk without aid 500 m”; and (2) a score of 6.0 on the EDSS, indicating “intermittent or unilateral constant assistance (cane, crutch or brace) required to walk 100 m with or without resting” [25], as well-known disability outcomes.

### 4.1. Standard Protocol Approvals, Registrations and Patient Consents

The study protocol was approved by the local Ethics Committee (SM.MP. 347., 3 February 2014) and was conducted in accordance with the ethical principles of the Declaration of Helsinki and with the appropriate national regulations. Patients provided written informed consent.

### 4.2. Statistical Analysis

To analyze the time to disability, a time to event variable was created using the time between time of first pregnancy and the follow-up date where EDSS 4.0 and/or EDSS 6.0 were first reached. The time between first pregnancy and the date of most recent follow-up visit was used for subjects who did not reach EDSS 4.0 and/or EDSS 6.0. Demographic and disease characteristics were compared between the monoparous and multiparous groups, using Student’s *t*-tests and chi-square tests as appropriate. A Mann–Whitney U-test was used to compare groups for those variables with non-parametric data distributions. Kaplan–Meier curves were calculated to determine the unadjusted relationship between offspring number and disease progression; in terms of reaching EDSS 4.0 and EDSS 6.0. Log-rank statistics were used to assess differences between parity groups. This was followed by Cox proportional hazard regression models, which allowed for adjustment of the confounding factors. To assess for multicollinearity, the variable inflation factor (VIF) for each predictor was determined. We considered a two-sided *p*-value of <0.05 as statistically significant. Data were analyzed using Statistical Package for Social Sciences (SPSS) Version 22 (IBM SPSS Statistics 22, © IBM, Armonk, NY, USA).

## 5. Conclusions

Gathering information on pregnancy-related issues is of crucial importance for the counseling of wwMS. Pregnancy-related issues are not related only to the potential role of pregnancy on disease re-activation after delivery, but they are more and more depending on drug treatments. A number of disease-modifying drugs (DMDs)are available and other are under development to treat RR forms of MS. None of these agents is approved for use in pregnancy [26]. The timing of treatment *vs.* conception and the risk of drug pregnancy exposures are frequent discussion topics when caring for wwMS and their partners. Moreover, wwMS facing a possible pregnancy want to know its effects on long-term disability and whether they may have more than one child along their disease course. Various studies have shown no overall adverse effect of pregnancy on the prognosis of long-term disability in MS. Weinshenker *et al.* [21] analyzed the effect of pregnancy on long-term disability in MS. They found no association between disability and the total number of term pregnancies, timing of pregnancy and onset of MS or either onset or worsening of MS in relation to pregnancy in 185 women ascertained through a retrospective population-based survey in Canada. Roullet *et al.* [19] described a total of 49 pregnancies after MS onset in 317 wwMS. In an average follow up of 100 months, 21 wwMS had one pregnancy, eight had two and four had three pregnancies. They found that increasing disability (assessed by EDSS) was not related to pregnancy, even when prognostic variables, such as age at onset and duration of MS, were taken into account. Whether pregnancies can modulate MS course should be assessed in prospective studies in which patients would receive baseline information before deciding to start a pregnancy; and the determinants of their choice would be collected before onset of pregnancy. More studies are needed. The drop in relapse rates during pregnancy and its reported increase postpartum coincide with the rise and fall of sex steroids, including estrogens. In fact, estrogen levels are highest during late pregnancy and dramatically decrease after delivery [17].

Autoimmune diseases are more prevalent in women compared to men, an effect that is likely influenced by sex hormones, such as estrogens. The exact role that estrogens play in MS disease course is not fully understood. Estrogens have both immunological and neurological effects (see elsewhere, [27,28]). Briefly, during pregnancy, there is a shift towards anti-inflammatory Th2 lymphocytes and corresponding cytokine production, such as IL-4 andIL-10. The increased risk in relapses postpartum may be due to the maternal shift back towards a Th1 milieu [28]. Similar beneficial outcomes of pregnancy have also been reported in other Th1-mediated diseases, such as rheumatoid arthritis [28].

Studies using hormones in human subjects with MS are limited, but it was found that non-pregnant wwMS treated with oral estrogens showed a reduction in the number and volume of gadolinium-enhancing lesions [29]. Clinical trials using sex steroids are currently underway [29,30]. An important confounding factor in pregnancy and MS research is the probable bias of wwMS with severe disease symptoms bearing fewer children due to reduced reproductive activity or the fear of not being able to take care of a child [29], while wwMS with a less severe disease course might choose to become pregnant. In this regard, we found that multiparous groups of wwMS had a significantly lower level of disability at the moment of first pregnancy (1.2 *vs.* 1.7, *p* < 0.05). This is consistent with the described inverse trend between disability (assessed by EDSS) and number of births that exists in the literature [12].

We stated that it is unlikely that offspring number have an influence on the long-term prognosis for wwMS. The study has several limitations, such as the small sample size, the lack of specific data for the temporality of onset and pregnancy and the lack of a control group of no parous wwMS. One potential bias in interpreting our results could be that we cannot exclude whether the higher EDSS in monoparous wwMS was the reason why they had only one child. This could be due to the fact that wwMS with a worse form of MS could be scared about their disease and their capacity to handle more than one child. Future research should check for this potential bias.

We are conducting further data collection for an in-depth study to ascertain disability over time pointedly related to the temporal relationship between reproductive events and MS symptom onset and/or diagnosis, as well as pre- and post-partum DMD’s use.

Another limitation may be related to the recording of time to reach EDSS 4.0 and 6.0. In this study, measurements of EDSS were captured at follow-ups, and the exact date of reaching the disability milestone may be slightly different.

Further studies are needed to clarify the precise factors that could explain the potential role of pregnancy in modulating the disability’s accrual in wwMS.
